# Molecular subtypes of osteosarcoma classified by cancer stem cell related genes define immunological cell infiltration and patient survival

**DOI:** 10.3389/fimmu.2022.986785

**Published:** 2022-08-19

**Authors:** Lei Guo, Taiqiang Yan, Wei Guo, Jianfang Niu, Wei Wang, Tingting Ren, Yi Huang, Jiuhui Xu, Boyang Wang

**Affiliations:** ^1^ Musculoskeletal Tumor Center, Peking University People’s Hospital, Beijing, China; ^2^ Beijing Key Laboratory of Musculoskeletal Tumor, Beijing, China

**Keywords:** osteosarcoma, cancer stem cells, cancer stemness, molecular subtype, tumor microenvironment, drug

## Abstract

Recent studies have shown that tumor stemness has biological significance in tumorigenicity and tumor progression. However, the characteristics of TME immune infiltration in osteosarcoma mediated by the combined effects of multiple cancer stem cell-related genes remain unknown.

**Methods:**

In this study, we identified different cancer stem cell-associated subtypes in osteosarcoma based on 25 cancer stem cell-associated genes by consensus clustering analysis, and we comprehensively evaluated the association between these subtypes and immunocytes infiltration in the TME. The cancer stem cell (CSC) score was constructed to quantify the stemness of individual tumors.

**Results:**

We performed a comprehensive evaluation of 218 osteosarcoma patients based on 25 cancer stem cell-related genes. Three different cancer stem cells related subtypes were identified, which were related to different biological processes and clinical outcomes. The three subtypes have different TME cells infiltrating characteristics, and CSC Cluster A had a higher level of immunocyte infiltration compared to CSC Cluster B and C. We constructed a scoring system, called the CSC score, to assess the stemness of individual patients. Then we found that the prognosis of patients was predicted by CSC score, and patients with low CSC score had prolonged survival. Further analyses showed that low CSC score was correlated with enhanced immune infiltration. CSC score may predict the effect of immunotherapy, and patients with low CSC score may have better immune response and clinical prognosis.

**Conclusions:**

This study demonstrates that there could be three cancer stem cell-associated subtypes in osteosarcoma and that they were associated with different patient prognosis and TME immune infiltration characteristics. CSC score could be used to assess the stemness of individual patients, improve our comprehension of TME characteristics, and direct more effective immune therapy.

## Introduction

Osteosarcoma (OS) originates from mesenchymal tissue and is one of the most common malignancies of bone tissue. OS is highly malignant, rapidly progressive, and highly susceptible to postoperative recurrence. In particular, OS that occurs in adolescence has a very high rate of disease progression. The 5-year survival rate for early OS patients is 40-60%, while only 5-20% for advanced OS patients (1). Currently, OS is mainly treated by surgery combined with neoadjuvant chemotherapy, but it does not completely solve the problem of distant metastasis and postoperative recurrence in OS patients. The root cause of the poor prognosis of OS is that the current treatment measures cannot remove the remaining tumor cells and eventually lead to the recurrence and metastasis of the tumor (2). Therefore, in order to improve patient survival, there is an urgent need to investigate the pathogenesis of osteosarcoma, to identify important targets that regulate the initiation and progression of osteosarcoma, and to assess their potential therapeutic value, which will bring new light to improve the overall prognosis of osteosarcoma.

Cancer stem cell (CSC) is commonly defined as tumor cell with stem cell-like characteristics. The presence of such cells will likely lead to heterogeneity within the tumor (3). Similar to normal stem cell, CSC has self-renewal potential and differentiation ability, and they can expand by symmetrical or asymmetrical divisions (4, 5). CSC expands in a symmetrical division. Excessive growth of CSC will eventually lead to tumor formation (6). Similarly, CSC plays significant roles in tumor metastasis (7–9) and chemotherapy resistance (10–14). CSC shows variability in different cancers, and CSC differs genetically and phenotypically (15). Since CSC has been shown to cause tumor initiation as well as recurrence, the search for specific markers of CSC is particularly important (16). Kevin et al. examined and compared the expression of various CSC markers such as ABCB1, ABCG2, ALDH1A1, CD24, and CD44 in tumors and adjacent normal tissues using publicly available databases and found that most CSC markers were more highly expressed in tumors (17). Kevin et al. found that the CSC marker CD44 plays an important role in tumor metastasis, drug resistance, immune evasion, and epithelial mesenchymal transition (18). However, since most of the markers specific to CSC are also present in adult tissue resident stem cell populations, human embryonic stem cells (hESC) or adult tissues, their clinical application is still very limited (16). The highly aggressive and chemotherapy resistance of CSC leads to more challenging tumor treatments (19–21). In recent years, an increasing number of studies have attempted to treat cancer by targeting CSC-associated drug resistance and metastasis (22, 23). Ramesh et al. summarized the role of different signaling pathways in breast CSC and proposed different therapeutic strategies to target CSC (24).

Immunotherapy to destroy tumor cells by identifying immune infiltration in the tumor has become an effective treatment for many advanced cancers (25). Immunotherapy can activate anti-tumor immunity and improve the condition of the TME. TME is a complex and diverse dynamic system composed of multiple immunocytes, cytokines and stromal cells, which is often considered to be immunosuppressive (26). TME serves as a physical environment that supports the development of cancer cells, so exploring its phenotypic and functional heterogeneity will have important implications for the treatment of cancer (27). Both immune evasion and CSC is thought to mediate tumor growth and metastasis, thus exploring the interaction between immunocytes and CSC in TME has a significant role in improving immunotherapy. Notably, Miranda et al. revealed that high stemness is associated with poor immune infiltration in 21 malignancies, demonstrating a potential interaction between CSC and immunocytes (28). Tumor-associated macrophages (TAMs) are tumor-infiltrating myeloid cells. These cells are capable of functional and morphological alterations when affected by the tumor microenvironment. Several studies have revealed the complexity of crosstalk between CSC and TAMs, confirming that CSC is critical for recruitment with TAMs and that CSC may influence the polarization state of TAMs (29–32). Wei et al. discovered that CSC in malignant gliomas promote the survival of TAMs by secreting WISP1 (30). Similarly, Wen et al. demonstrated that CSC in glioblastoma multiforme can influence TAMs polarization and also recruit TAMs by secreting POSTN (31). Karina et al. found that CD44 could mediate the regulation of TAM for tumor stem cells *via* the PI3K-4EBP1-SOX2 pathway (32). CD8+ T cells have key roles in tumor immunity, and CSC interacts with CD8+ T cells in two main ways: CSC evades CD8+ T cell-mediated death (33) and CSC inhibits the antitumor immunity of CD8+ T cells (34, 35). Yu et al. found that CSC evaded cytotoxic T cell killing through TGFb-dependent upregulation of CD80 in murine epidermal squamous cell carcinoma (33). Jun et al. found that CSC in Glioblastoma multiforme inhibited T cells activation and proliferation, and triggered T cells apoptosis (35). Similarly, several studies have confirmed the correlation between CSC and tumorigenic dendritic cells (DCs) (36–38). Most studies have focused on individual CSC-associated genes and one type of immune cell, however CSC has the complexity of high synergistic effects of multiple genes. Therefore, exploring the infiltrative properties of TME cells mediated by the combined action of multiple CSC-related genes will help enhance our comprehension of osteosarcoma TME.

In this study, we included transcriptomic data and clinical information from a total of 218 osteosarcoma patients and identified three distinct CSC clusters in osteosarcoma. We evaluated the three CSC clusters comprehensively and systematically analyzed the association between different subtypes and TME. In addition, we constructed a CSC score system for quantifying CSC-related modalities in individual osteosarcoma patients, which was validated in multiple independent datasets. These results suggested the important roles of the combined action of multiple CSC-related genes in osteosarcoma TME.

## Materials and methods

### Data collection and preprocessing

RNA expression data and clinical information of osteosarcoma patients were downloaded from TARGET (https://ocg.cancer.gov/programs/target) and GEO (https://www.ncbi.nlm.nih.gov/geo/) databases. The following were the inclusion criteria: (a)osteosarcoma samples with gene expression matrix; (b)samples with clinical information such as age, gender, survival time, survival status, and whether metastasis occurred; (c)samples with expression values for more than half of the genes. Based on the above criteria, 4 eligible osteosarcoma cohorts (GSE21257, GSE39055, GSE16102, and TARGET-OS)were collected for further analysis. The batch effect of non-biotechnical bias was corrected using the “ComBat” package. The cohorts GSE21257, GSE39055 and GSE16102 were merged into the meta-cohort. 331 cancer stem cells-associated genes (CSCRGs) were obtained from the molecular marker database, of which 228 genes were expressed in the TARGET-OS cohort and meta-cohort. In the TARGET-OS cohort, we selected 25 CSCRGs for further studies using univariate COX analysis (P<0.05) (**Supplementary Tables 1** and **2**).

### Identification of molecular subgroups and calculating DEGs

The consensus clustering was performed using the “ConsensusClusterPlus” package based on the expression matrix of the 25 CSCRGs (39). Differentially expressed genes (DEGs) between three clusters were analyzed using ‘limma’ package with the cutoff criteria of P < 0.05.

### Functional analyses and TIME evaluation

The Gene Ontology (GO) enrichment was performed using the “clusterprofiler” package. The geneset “h.all.v7.5.1.symbols” was obtained from MSigDB. Mariathasan et al. had constructed a geneset in which genes related to certain biological processes were stored (40). Based on the above gene sets, we performed Gene set variation analysis (GSVA) by using the “GSVA” and “limma” packages to show alterations in signaling pathways among three clusters (41). Adjusted P value less than 0.05 was defined as statistically significant. The ESTIMATE algorithm was used to calculate the immune score, stromal score and tumor purity.

### Estimation of TME cell infiltration

Cohorts of 23 immune infiltrating cells and 13 immune-related functions were obtained (42). And the score calculated with ssGSEA was used to express the relative abundance of different immunocytes and immune-related functions in every case (43). The abundance of six immunocytes were analyzed using the TIMER algorithm.

### Construction of the CSC score

In order to quantify the stemness of individual tumors, we constructed a score system called CSC score, which was constructed in the following steps. We selected overlapping DEGs found in distinct CSC clusters and performed prognostic analyses of individual genes by using univariate Cox regression. Extraction of genes with remarkable prognosis was used to construct the CSC score by principal component analysis (PCA). Principal component1 and Principal component2 were used in the construction of the CSC score. We also defined CSC score using a similar approach from previous studies (44, 45). CSC score =ΣPC1_i_ + PC2_i_, i is the expression of genes associated with the CSC phenotype. We stratified the tumors into CSC score low and high subgroups using the surv-cutpoint function in the ‘survival’ package.

### Drug sensitivity assessment

We used the ‘pRRophetic’ package to assess the sensitivity to different CSC clusters to small molecule drugs. In addition, the CellMiner database was utilized to assess the association between CSCRGs and different drugs (46).

### Calculation of mRNAsi

Based on one-class logistic regression (OCLR) algorithm, the stemness index model trained from the Progenitor Cell Biology Consortium database was used to calculate tumor stemness (47, 48). The stemness index can be used to measure how similar tumor cells are to stem cells, with stemness index being a value between 0 (lowest) and 1 (highest). The closer the stemness index is to 1, the stronger the stem cell properties. We calculated transcriptome feature scores for the cohorts using the same Spearman correlation.

### Clinical samples and immunohistochemistry

A total of 10 OS tissues were collected, all from Peking University People’s Hospital. The samples were examined by three experienced pathologists. All patients provided informed consent, and the study protocol was approved by the Ethics Commttee of Peking University People’ s Hospital (2019PHB198-01). Immunohistochemical examination was performed with *MEF2C* antibody (10056-1-AP, proteintech).

### Cell culture and transfection

Human osteosarcoma cell line (143B) was purchased from the American Type Culture Collection (ATCC). 143B cells was cultured in DMEM (Gibco) containing 10% fetal bovine serum (FBS, Gibco) and 1% penicillin and streptomycin (Gibco). 143B cells were cultured in a humidified incubator with 5% CO2 at 37°C. Si-*MEF2C* (Suzhou, China, sequences: 5’GACAAGGAAUGGGAGGAUA3’) and GP-transfect-Mate (Suzhou, China) were used for transfection.

### Sphere formation assay

143B cells were inoculated at a density of 1000 cells/well in six-well ultra-low attachment plates (Corning). And the cells were cultured in DMEM/F12 medium (Gibco) containing N2 medium (Invitrogen), human EGF (20ng/ml, PeproTech) and human bFGF (20ng/ml, PeproTech) for 14 days. Spheres were observed in size and the number of spheres formed was calculated.

### Cell adhesion assay

50 µl of vitronectin (PeproTech) or fibronectin (Biocoat) was added to each well of a 96-well plate and incubated overnight at 4°C. Unbound proteins were washed with PBS and closed with PBS containing 2% BSA for 2 hours at 37°C. 143B cells were inoculated at a density of 10000 cells/well in 96-well plate (Corning). And the cells were cultured in DMEM (Gibco) for 1 hour. Unbound cells were washed with PBS, fixed with 4% paraformaldehyde and stained with 0.1% crystal violet. Finally, the number of adherent cells was observed under the microscope.

### Statistical analyses

Statistical analyses were performed *via* R (version 4.1.2), and survival analyses were performed using the Kaplan-Meier method. The Student’s t test was used for normally distributed variables and the Wilcoxon rank sum test was used for non-normally distributed variables. The Kruskal-Wallis test and one-way ANOVA were used for the non-parametric and parametric methods, respectively (49). Correlations coefficients between the expression of CSCRGs and the TME infiltrating immunocytes were calculated by Spearman analysis. We used univariate Cox regression analysis to compute the hazard ratio (HR) of CSCRGs and CSC-related signature genes. To verify whether the constructed risk scores can be used as an independent prognostic factor independently of other clinical traits. Univariate and multivariate COX analyses of patients’ age, gender, presence of metastasis, and CSC score were performed. The predictive performance of the CSC score was assessed by using receiver operating characteristic (ROC) curves, and the area under the curve (AUC) was calculated by the ‘timeROC’ package. P-value was bilateral and P < 0.05 was defined as statistically significant difference.

## Results

### Landscape of 25 cancer stem cells related genes in osteosarcoma

**Figure 1:**
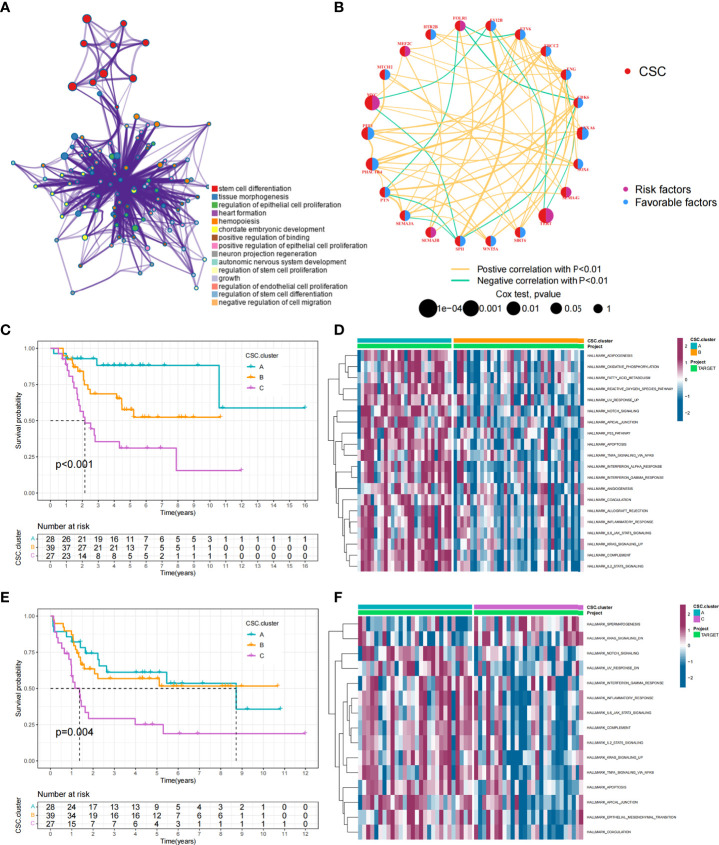
CSC-related subtypes and biological characteristics of each subtype. **(A)** Metascape enrichment network. Color codes indicate different clusters. **(B)** Interaction of 25 CSCRGs in osteosarcoma. The red color on the left half of the circle represented the type of gene. The blue color on the right half of the circle indicated favorable factors; the purple color on the right half of the circle indicated risk factors. The size of the circles was determined by the p-value, representing the impact of each gene on the prognosis of patients. The lines between genes indicated their interactions, positive correlations were shown in yellow and negative correlations were shown in green. **(C, D)** Kaplan-Meier curves of overall survival **(C)** and event-free survival **(D)** for 94 osteosarcoma patients in TARGET cohort with different CSC cluster, including 28 cases in CSC cluster A, 39 cases in CSC cluster B, and 27 cases in CSC cluster C (Log-rank test). **(E, F)** The heatmap visualized the results of the GSVA enrichment analysis in the TARGET cohort, and purple represents activated pathways and blue represents inhibited pathways.

In this study, we finally selected 25 cancer stem cells related genes (CSCRGs) by univariate COX analysis in TARGET-OS cohort and later investigated the role of these genes in osteosarcoma. Metascape analysis and GO enrichment analysis were performed on 25 CSCRGs, which were seen to be enriched in multiple stemness-related regulatory pathways, and the results were shown in **Figure 1A** and **Figure S1A**. In addition, Spearman analysis was used to assess the relevance between 25 CSCRGs (**Figure S1B**). COX regression analysis was used to analyze the relationship of 25 CSCRGs with patient prognosis in osteosarcoma. The forestplot showed that *FOLR1, SEMA3B, SEMA4G, MYC, OVOL1 and MEF2C* were considered as risk factors (**Figures S1C, D**). The above analyses showed 25 CSCRGs played important roles in the development and progression of osteosarcoma.

### Cancer stem cells related subtypes mediated by 25 CSCRGs

Data of 94 osteosarcoma patients in the TARGET-OS cohort were used for analysis. The network showed the interactions of the 25 CSCRGs and their prognostic significance for osteosarcoma patient (**Figure 1B**). The results indicated that these 25 CSCRGs were mutually regulated and played crucial roles in the development of osteosarcoma. We utilized consensus clustering algorithm to stratify samples into distinct CSC clusters based on the expression of the 25 CSCRGs. Consequently, we identified three distinct clusters, including 28 samples in cluster A, 39 samples in cluster B and 27 samples in cluster C (**Figures S2A, B**, **Supplementary Tables 3**, **4**). Prognostic analysis for these three clusters indicated that CSC cluster A showed an excellent survival advantage, while CSC cluster C had the worst prognosis in TARGET-OS cohort (**Figures 1C, D**). We also noted that there were remarkable differences in expression levels of the 25 CSCRGs between distinct clusters (**Figures S2C, D**). *ANXA6, ENG, EVI2B, SEMA3E*, and *SPI1* were significantly elevated in CSC cluster A, *CDK6, ETV6, MYC, PHACTR4, PTN, SEMA3A*, and *SOX4* were evidently increased in CSC cluster B, and *SEMA4G* was evidently increased in CSC cluster C (**Figures S2C, D**).

### TME cell infiltration characteristics in distinct cancer stem cells related subtypes

**Figure 2:**
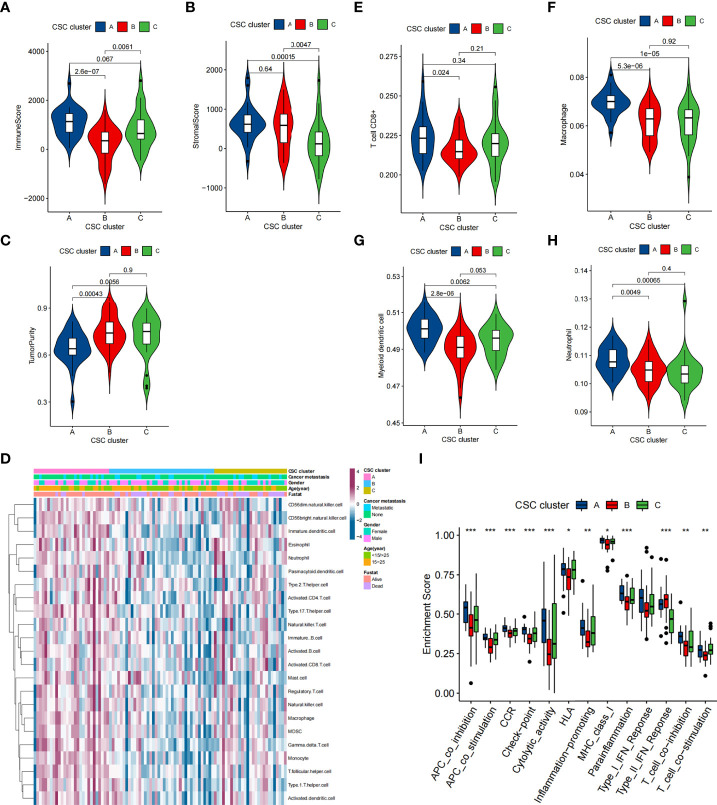
TME characteristics in distinct CSC clusters in the TARGET cohort. **(A–C)** Immune score **(A)**, stromal score **(B)** and tumor purity **(C)** of three CSC clusters were analyzed and plotted. The whisker contained 1.5 times the interquartile range. The bottom and top of the box indicate the 25th and 75th percentiles, and the thick line indicates the median value. **(D)** The heatmap used to visualize the infiltration of 23 immunocytes in three CSC clusters. CSC cluster, age, gender, patient survival status and tumor metastasis status were annotated. Purple represented high immune infiltration and blue represented low immune infiltration. **(E–H)** CD8+ T cells, neutrophils, macrophages and myeloid dendritic cells abundance in three CSC clusters was calculated using TIMER2.0. **(I)** Differences in the immune-related functions between three CSC Clusters. The bottom and top of the box indicate the 25th and 75th percentiles, and the thick line indicates the median value. (*P < 0.05; **P < 0.01; ***P < 0.001).

To explore the biological behaviors underlying these different CSC clusters, we performed GSVA enrichment analysis (**Figures 1E, F**). The results showed that CSC cluster A was markedly abundant in immune activation-related processes, such as complement, inflammatory and interferon gamma response, and the TNFA pathway, KARS pathway and apoptosis pathway were also enriched in CSC cluster A. Based on the above findings, we presumed that the better prognosis of CSC cluster A might be related to its high immune infiltration. Furthermore, we quantified the stromal score, immune score and tumor purity for the three clusters using ESTIMATE algorithm. The analysis showed that CSC cluster A had the highest immune score and stromal score, followed by CSC cluster B and C (**Figures 2A, B**). Conversely, CSC cluster B and C had higher tumor purity compared to CSC cluster A, suggesting that tumors in CSC cluster A were surrounded by more non-tumor components (immunocytes and stromal cells) (**Figure 2C**). In addition, a heat map was built by ssGSEA to visualize and compare the abundance of 23 immunocytes under different clusters (**Figure 2D**). The great majority of immunocytes such as anti-tumor lymphocyte cell subpopulations and NK T cells were mainly enriched in the CSC cluster A. We further described the immune infiltration profile using TIMER2.0 and observed consistent results (**Figures 2E–H**). The results showed that neutrophils, macrophages and myeloid dendritic cells were mainly enriched in the CSC cluster A, while CD8+ T cells were enriched in CSC cluster A and C. We also calculated 13 immune-related function scores using ssGSEA, and the results suggested that majority of immune-related functions were enriched in CSC cluster A (**Figure 2I**). Curiously, CSC cluster C also had a higher infiltration of immune cells but did not show the same survival advantage. DCs have been shown in previous studies to be responsible for antigen presentation and initial T cell activation, bridging innate and adaptive immunity, and their activation is dependent on high expression levels of MHC molecules, co-stimulatory factors and adhesion factors. Therefore, we compared the expression of MHC molecules, co-stimulatory molecules and adhesion molecules in the three CSC subtypes and found that most molecules including *CD40, CD80, CD86, HLA-C, HLA-DMC, HLA-DMB, HLA-DPB1, HLA-DRA, HLA-E*, and *ICAM1* were significantly elevated in CSC cluster A (**Figure S2E**). Thus, we hypothesize that although CSC cluster C had a higher immune infiltration, its antigen-presenting ability and ability to activate DCs were weaker compared to CSC cluster A. Therefore, CSC cluster C had a poorer survival prognosis compared to CSC cluster A. The results from GSVA analysis demonstrated that Pan-F-TBRS, antigen processing machinery, immune checkpoint, and CD8 T effector were enriched in CSC cluster A further corroborating our hypothesi (**Figure S2F**). Taking into account that PD-L1 is a proven biomarker to predict immunotherapy response (37), we identified a significant upregulation of PD-L1 expression levels in CSC cluster A (**Figure S2G**). Based on these findings, we identified three subtypes with distinct immune infiltration characteristics.

**Figure 3:**
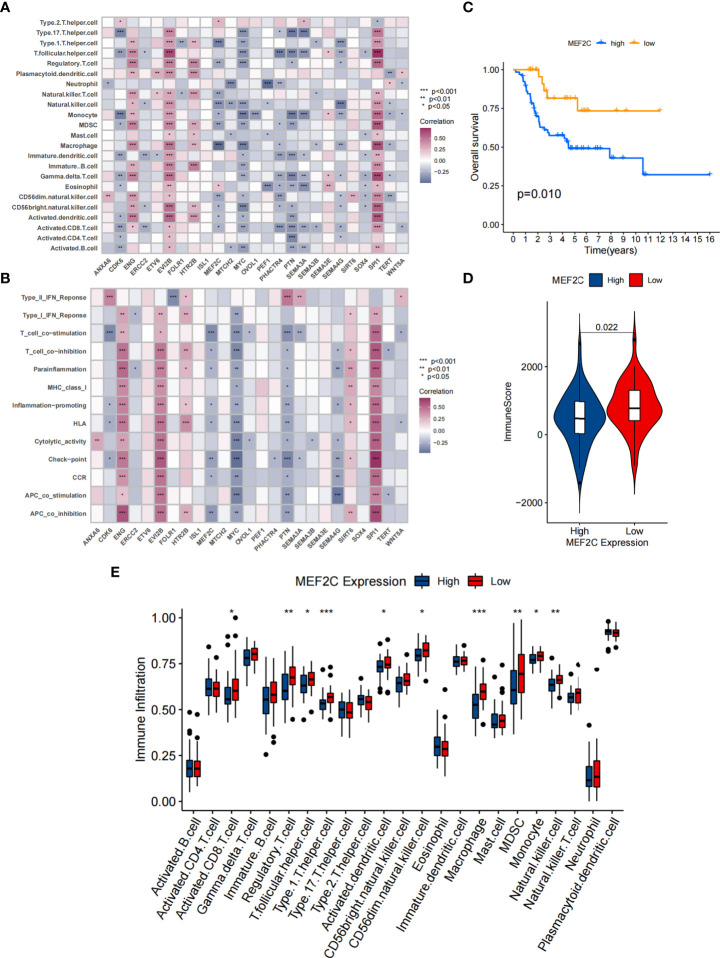
Correlation between TME infiltration and 25 CSCRGs and the relationship between MEF2C and immune infiltration. **(A)** The correlation between each immunocyte and each CSCRG by spearman analysis. Purple indicates positive correlation and blue indicates negative correlation. **(B)** The correlation between each immune-related function and each CSCRG using spearman analysis. Purple indicates positive correlation and blue indicates negative correlation. **(C)** Kaplan-Meier curves were used to analyze the survival of the high and low expressing MEF2C patient group. **(D)** Comparison of immune score between high and low MEF2C expressing subgroups. **(E)** Difference in the abundance of each immunocyte between MEF2C high expression and low expression subgroups. (*P < 0.05; **P < 0.01; ***P < 0.001).

In addition, Spearman analysis was used to assess the specific relationship between the 25 CSCRGs and immunocyte infiltration or immune-related function (**Figures 3A, B**). High expression of *EVI2B, ENG* and *SPI1* was markedly associated with enhanced immunocyte infiltration and immune-related function, whereas *MEF2C, PHACTR4, MYC, PTN, SEMA3A* and *SEMA4G* high expression showed a negative correlation with the immunocyte infiltration and immune-related function level. Among the 25 CSCRGs, we focused on *MEF2C*, which was found to be markedly negatively correlated with substantial immune infiltration and immune-related function levels and the expression high of *MEF2C* was significantly negatively correlated with patient prognosis (**Figure 3C**). We firstly compared the overall level of immune cell infiltration in patients with high and low *MEF2C* expression. Patients with low *MEF2C* expression had higher immune scores, indicating that TME immune cell infiltration was markedly increased in patients with low *MEF2C* expression (**Figure 3D**). We then compared the differences in 23 immunocytes between the two subgroups with low and high *MEF2C* expression (**Figure 3E**). We found that high *MEF2C* expression was remarkably negatively associated with the levels of infiltration of multiple immune cells, including regulatory T cells, T follicular helper cells, type 1 T helper cells, macrophages, MDSCs, natural killer cells, activated DCs and CD8+ T cells (**Figure 3E**). DCs are responsible for antigen presentation and initial T cell activation, bridging the gap between innate and adaptive immunity [52]. According to these findings, we hypothesize that *MEF2C* may inhibit the cytotoxic T lymphocytes and activated DCs, thereby hindering the intratumoral anti-tumor immune response. Based on the above findings, we speculated that the expression of *MEF2C* could influence the prognosis of patients by affecting the infiltration of multiple immune cells.

### Cancer stem cells related subtypes in GSE21257 cohort

**Figure 4:**
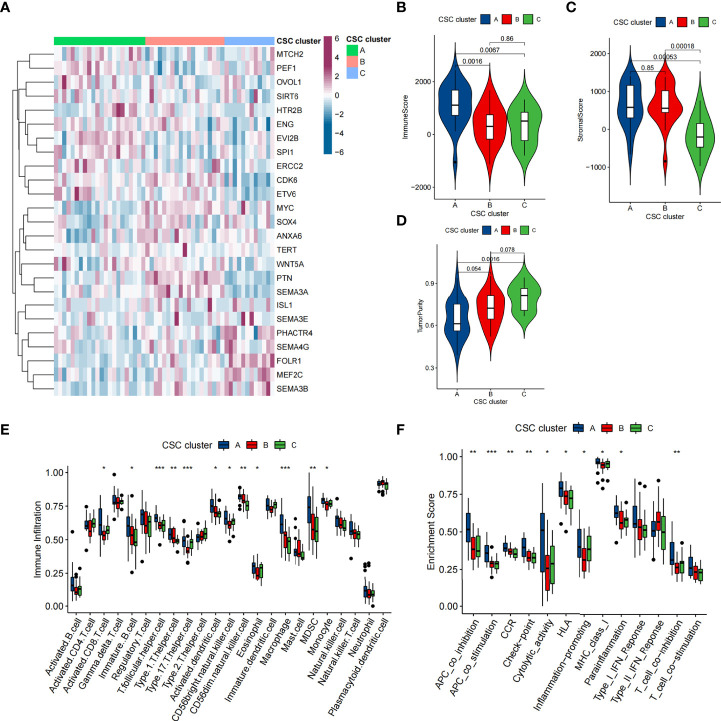
TME characteristics in distinct CSC clusters in GSE21257 cohort. **(A)** Unsupervised clustering of 25 CSCRGs in the GSE21257 cohort. CSC cluster, age and gender were annotated. Purple indicated high expression of the gene and blue indicated low expression of the gene. **(B–D)** Immune score **(B)**, stromal score **(C)** and tumor purity **(D)** of three CSC clusters were analyzed and plotted. **(E)** Difference in the abundance of each immunocyte between three CSC clusters. **(F)** Differences in the immune-related functions between three CSC Clusters. (*P < 0.05; **P < 0.01; ***P < 0.001).

To further confirm that the typing based on 25 CSCRGs was also applicable to other datasets, we performed validation with the GSE21257 cohort. Similar to the clustering results of the TARGET-OS cohort, unsupervised clustering also revealed three completely different clusters of the 25 CSCRGs in the GSE21257 cohort (**Supplementary Table 5**, **Figures S3A, B**). The mRNA expression of 25 CSCRGs was significantly different in the three cluster (**Figure 4A**). Prognostic analysis indicated that the survival of CSC cluster A was better than that in CSC cluster B and C (**Figure S3C**). We also quantified the stromal score, immune score and tumor purity for the three clusters using ESTIMATE algorithm. The results showed that CSC cluster A had the highest immune score compared to CSC clusters B and C (**Figure 4B**), and CSC clusters A and B had a higher stromal score compared to CSC cluster C (**Figure 4C**). And consistent with the previous result, CSC cluster A had lower tumor purity (**Figure 4D**). We then analyzed the differences in immune infiltration between the three clusters, and we found the vast majority of immunocytes such as anti-tumor lymphocyte cell subpopulations and NK T cells were largely enriched in the CSC cluster A (**Figure 4E**). And consistent with previous results, most immune-related functions were enriched in CSC cluster A (**Figure 4F**). Similarly, the results of gsva analysis remained consistent with previous ones, showing enrichment of CD8 T-effects, immune checkpoints and Pan-F-TBRS in the CSC cluster A (**Figure S3D**).

### Cancer stem cells phenotype-related DEGs in osteosarcoma

**Figure 5:**
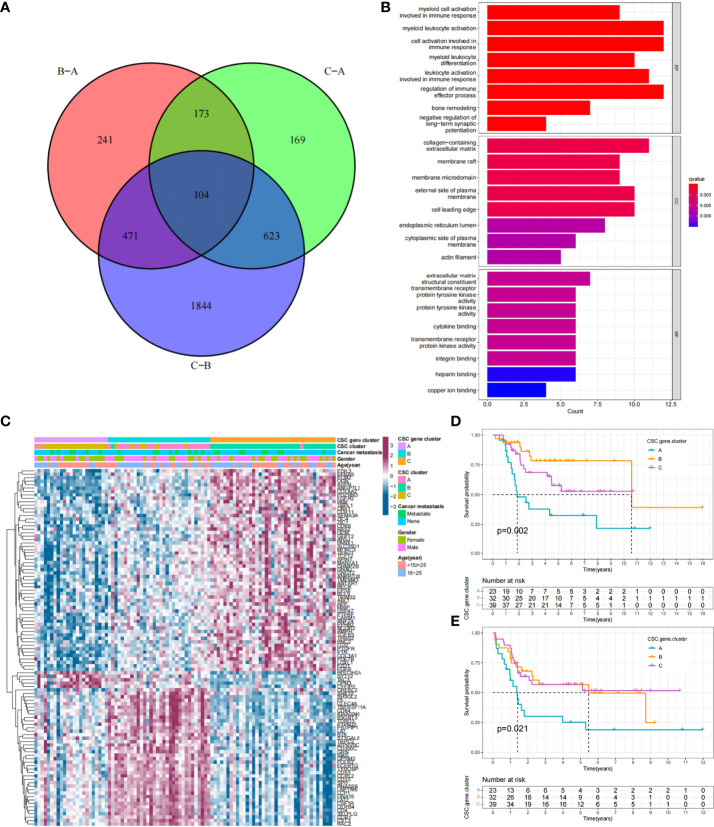
Functional annotation of DEGs and unsupervised clustering analysis based on DEGs. **(A)** Venn diagram showing the 104 CSC-related genes. B-A: DEGs between B and A; C-A: DEGs between C and A; C-B: DEGs between C and B, **(B)** GO enrichment analysis of the 104 CSC-related genes. The x-axis indicated the number of genes enriched. BP: biological process; CC: cellular component; MF: molecular function. **(C)** Unsupervised clustering of 104 CSC-related genes in the TARGET cohort. CSC gene cluster, CSC cluster, age, gender and tumor metastasis status were annotated. Purple indicated high expression of the gene and blue indicated low expression of the gene. **(D, E)** Kaplan-Meier curves of overall survival **(D)** and event-free survival **(E)** for 94 osteosarcoma patients in TARGET cohort with different CSC gene cluster, including 23 cases in CSC cluster A, 32 cases in CSC cluster B, and 39 cases in CSC cluster C (Log-rank test).

Previously, tumor samples were divided into three subtypes associated with CSC based on 25 CSCRGs, and to further explore the genetic alterations in these phenotypes, we determined 104 CSC-related DEGs using limma package in the TARGET-OS cohort (**Figure 5A**, **Supplementary Table 6**). We performed GO enrichment analysis on these DEGs, which were seen to be enriched in multiple stemness-related and immune regulation pathways (**Figure 5B**). Furthermore, the above analysis supported that DEGs were closely associated with tumor immunity and cancer stemness, and thus might be considered as CSCRGs. We performed consensus clustering analysis on the 104 CSC phenotype-related genes obtained to further validate this regulatory mechanism and obtained three gene clusters (**Figures S4A, B**). We called these clusters as CSC gene cluster A-C. These results suggested that three distinct CSC subtypes indeed existed in osteosarcoma (**Figure 5C**). Among the three CSC gene clusters, 25 CSCRGs were found to be significantly differentially expressed, which was consistent with the expected results (**Figure S4C**). Survival analysis further showed prognostic differences between three CSC gene clusters. CSC gene cluster B was shown to be related to better prognosis, while CSC gene cluster A was proven to be related to worse prognosis (**Figures 5D, E**). To explore the roles of the 104 genes in immune infiltration, we examined the differences in 23 TME immune cells in the three clusters and showed that the most immunocytes increased in CSC gene cluster B (**Figure S4D**). Similarly, the result suggested that most immune-related functions were enriched in CSC gene cluster B (**Figure S4E**). Meanwhile, we found that PDL1 expression was significantly upregulated in CSC gene cluster B compared with CSC gene cluster A (**Figure S4F**). The above results once again suggested that CSC-related genes played non-negligible regulatory roles in the formation of different TME landscapes.

### Construction of the CSC score and exploration of its clinical significance

**Figure 6:**
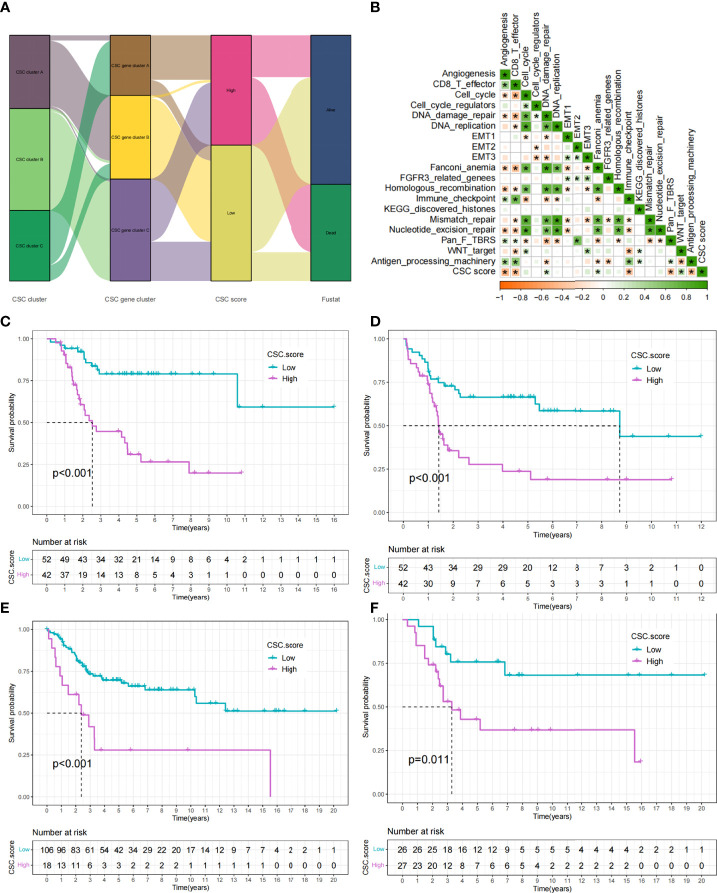
Construction of CSC score. **(A)** Alluvial diagram showing the changes of CSC cluster, CSC gene cluster, CSC score and patient survival status. **(B)** Correlations between CSC score and the certain gene signatures in TARGET cohort. Negative correlation was marked with yellow and positive correlation with green (*P < 0.05). **(C)** Survival analyses for high (42 cases) and low (52 cases) CSC score subgroups in TARGET cohort. **(D)** Event-free survival analyses for high (42 cases) and low (52 cases) CSC score subgroups in TARGET cohort. **(E)** Survival analyses for high (18 cases) and low (106 cases) CSC score subgroups in meta-cohort. **(F)** Survival analyses for high (27 cases) and low (26 cases) CSC score subgroups in GSE21257 cohort.

While previous studies have found important roles in prognosis and regulation of immune infiltration for CSC-related genes, these findings were only applicable to assess patient populations and could not be used to evaluate individual patients. Taking into account individual differences, we constructed a score system to quantitate CSC-related subtypes in single osteosarcoma patients based on the discovery of CSC-related signature genes, which we called CSC score. We constructed an alluvial diagram to illustrate the workflow of CSC score construction (**Figure 6A**). The result showed that CSC gene cluster A was related to higher CSC scores, and CSC gene cluster B was associated with lower CSC scores (**Figure S5A**). Notably, consistent with the expected results, CSC cluster B and C showed a higher CSC score than CSC cluster A (**Figure S5B**). We examined the relationship between CSC scores and certain biometric scores by Spearman analysis. The result showed that CSC score was negatively related to immune activation-related processes (**Figure 6B**). CSC score was also significantly negatively related to immune score (**Figure S5C**). Similarly, patients in the low CSC score subgroup had a higher degree of immune infiltration and were more enriched for immune-related functions compared to patients in the high CSC score subgroup (**Figures S5D, E**). The above analyses clearly indicated that low CSC score was remarkably correlated with immune infiltration. Based on the above findings, we concluded that the CSC score could better assess the CSC-related subtypes of individual tumors and further assess the tumor immune infiltration characteristics. Furthermore, we attempted to determine the value of CSC score in predicting patient prognosis. Patients were separated into low and high subgroups with a cutoff value of -0.3269, and patients with low CSC score had a better prognosis (**Figures 6C,D**). ROC curve analysis result verified the predictive advantage of the CSC score system (**Figure S6A**). We analyzed multiple clinical traits of patients using multivariate Cox regression and found that the CSC score system could potentially serve as an independent prognostic factor of osteosarcoma (**Figure S6B**). Futhermore, PD-L1 expression level was significantly higher in the group with low CSC score (**Figure S6C**). The constructed CSC scoring system was validated by meta-cohort, and patients with low CSC score indicated better prognosis (**Figure 6E**, **Supplementary Table 7**). To further validate the reliability of the CSC score system, we used a cohort (GSE21257) from the meta cohort to explore the association between CSC score and patient prognosis. Consistent with the results above, patients with low CSC score showed a significant survival advantage relative to patients with high score (**Figure 6F**). Similarly, ROC curve analysis result verified the predictive advantage of the CSC score system (**Figure S6D**). The above results strongly indicated that CSC scores could represent the CSC pattern of osteosarcoma patients and predict the prognosis of osteosarcoma patients.

**Figure 7:**
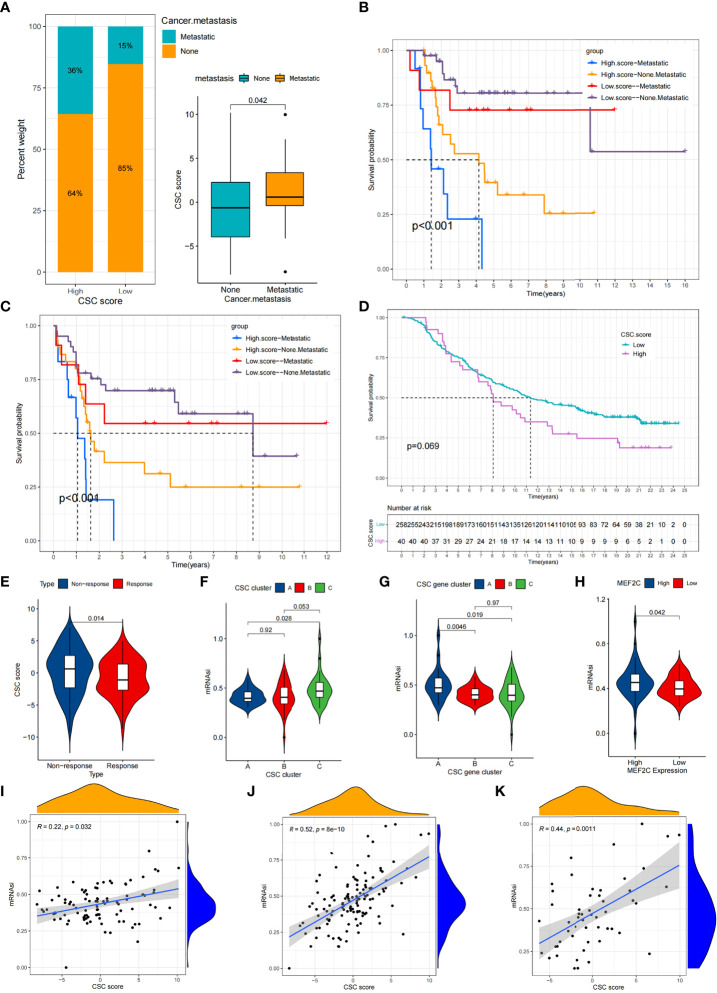
Comparison of CSC score between different clinical subgroups and relationship between mRNAsi and CSC score. **(A)** Comparison of CSC scores between tumor metastasis and tumor non-metastasis groups. **(B,C)** Kaplan-Meier curves of overall survival **(B)** and event-free survival **(C)** for 94 osteosarcoma patients in TARGET cohort with different tumor metastasis status. **(D)** Survival analyses for high (40 cases) and low (258 cases) CSC score subgroups in IMvigor210 cohort. **(E)** Comparison of CSC scores between patients who responded better to anti-PD-L1 therapy and those who responded less well. **(F)** Comparison of mRNAsi between three CSC clusters. **(G)** Comparison of mRNAsi between MEF2C high expression and low expression groups. **(H)** Comparison of mRNAsi between three CSC gene clusters. **(I–K)** CSC score and mRNAsi were significantly and positively correlated in the TARGET cohort **(I)**, meta cohort **(J)** and GSE21257 cohort **(K)**.

CSC is associated with a variety of clinical traits such as tumor metastasis. We compared the differences of CSC score between different clinical subgroups in the TARGET cohort. Accordingly, we found that tumor metastasis was significantly related to higher CSC score, implying that these patients were characterized by poor immune infiltration, with a poorer clinical outcome (**Figure 7A**). And there were no differences in scores between age and gender subgroups (**Figures S6E, F**). In particular, we found that low CSC score subgroup showed a significant survival advantage both in patients who had developed tumor metastases and in those who did not (**Figures 7B,C**). Additional results showed that the predictive ability of the CSC score was not interfered by whether the tumor had metastasized, and the low CSC score group consistently showed a significant survival advantage in both patients with and without metastasis (**Figures 7B, C**). These results suggest that the CSC score can also be used to assess certain clinical symptoms such as tumor metastasis in osteosarcoma patients. Considering the strong correlation between CSC score and immune response in the above results, we investigated whether CSC score could predict treatment response to anti-PDL1 in patients in an independent immunotherapy cohort. We performed a systematic search that resulted in the inclusion of an immunotherapy cohort: uroepithelial carcinoma intervening with atezolizumab (IMvigor210 cohort). The result indicated that patients with low CSC score scores gained a survival advantage (**Figure 7D**). And there was a significant therapeutic advantage of anti-PDL1 treatment in patients with low CSC scores (**Figure 7E**). The above results suggested that the CSC score might be used to assess the therapeutic response and clinical prognosis of immunotherapy.

### Exploring the correlation between CSC score and mRNAsi

Using the OCLR algorithm, the stemness index was computed for individual samples based on the gene expression profile of the patient. We compared the mRNAsi between different CSC clusters and showed that mRNAsi was significantly elevated in CSC cluster C (**Figure 7F**).Similarly, mRNAsi was significantly elevated in CSC gene cluster A (**Figure 7G**). We also found that patients with high *MEF2C* expression possessed higher mRNAsi (**Figure 7H**). Considering that mRNAsi represents the stemness of individual patients, we analyzed the correlation between CSC score and mRNAsi. The results revealed a significant positive relationship between CSC score and mRNAsi in the TARGET cohort (**Figure 7I**). However, the result showed a weak correlation between CSC score and mRNAsi (R=0.22). Therefore, unlike the previous result that the CSC score of CSC cluster A was significantly lower than cluster B, the mRNAsi of CSC cluster A was not significantly different from cluster B. Similarly, there was a significant positive correlation between CSC score and mRNAsi in both the meta cohort and the GSE21257 cohort (**Figures 7J, K**). The above results again demonstrated that the CSC score was a reliable scoring system that could represent the stemness of individual patients.

### Drug sensitivity profifiles of distinct cancer stem cells related subtypes

**Figure 8:**
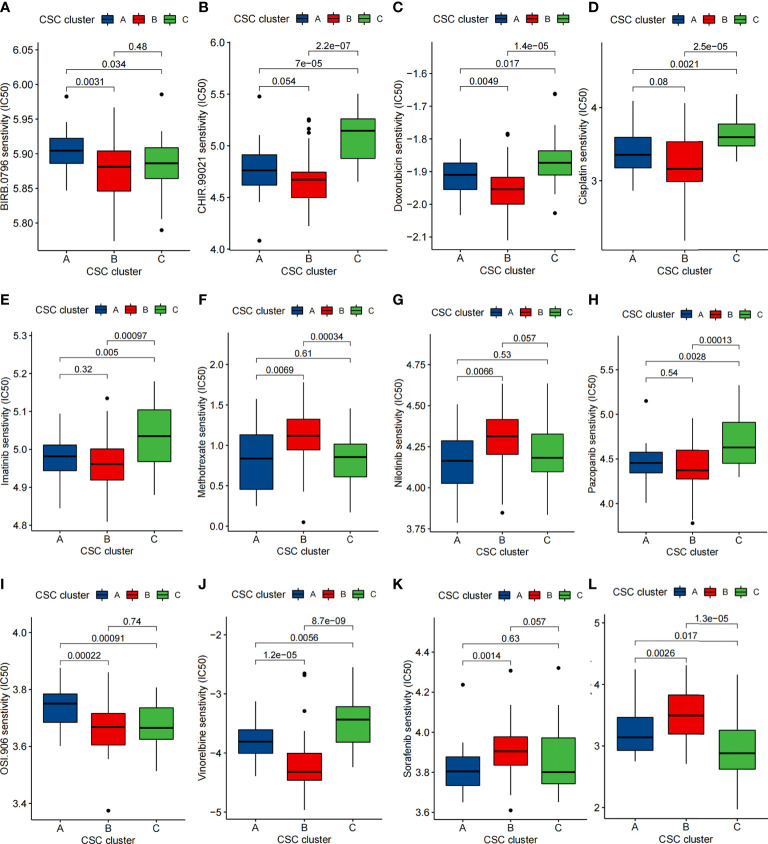
Comparison of drug sensitivity. **(A–L)** Comparison of IC50 of small molecule drugs between three CSC clusters.

We performed a drug sensitivity analysis and identified 74 small molecule drugs that may be used in the treatment of osteosarcoma (**Supplementary Table 8**). Sensitivity of 12 drugs in different CSC clusters was depicted in **Figures 8A-L**. The results showed that CSC cluster A was sensitive to BIRB.0796 and OSI.906, while CSC clutser B was sensitive to Methotrexate, Sorafenib, Sunitinib and Nilotinib, and CSC clutser C was more sensitive to Cisplatin, Doxorubicin, Imatinib, CHIR.99021, Pazopanib and Vinorelbine were more sensitive (**Figures 8A-L**). We then evaluated the relationship between 25 CSCRGs expressions and drug sensitivity using the CellMiner database (**Figure S7**, **Supplementary Table 9**). The above findings indicated that exploring CSC subtypes in osteosarcoma patients could be used to guide the clinical use of drugs.

### Immunohistochemical detection of *MEF2C* expression distribution

**Figure 9:**
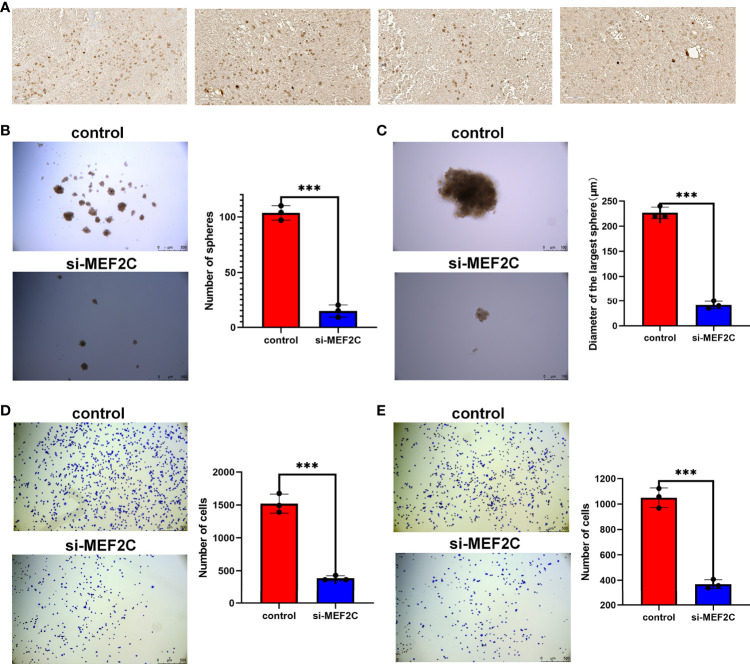
Immunohistochemical analysis of *MEF2C* expression in OS tissues and effect of *MEF2C* on the stemness of osteosarcoma cells. **(A)** Immunohistochemical analysis of *MEF2C* expression in OS tissues. **(B)** Comparison of the number of spheres between control and si-*MEF2C* groups. (***P < 0.001). **(C)** Comparison of the size of the largest of sphere between control and si-*MEF2C* groups. **(D)** Comparison of cell adhesion to vitronectin between control and si-*MEF2C* groups. **(E)** Comparison of cell adhesion to fibronectin between control and si-*MEF2C* groups.

Given that *MEF2C* was found to affect prognosis, immune infiltration and stemness in osteosarcoma patients in our previous study, we verified the expression of *MEF2C* in clinical tissues of osteosarcoma patients by immunohistochemical experiments. The results showed that *MEF2C* was significantly expressed in clinical tissues of osteosarcoma patients (**Figure 9A**).

### *MEF2C* affected the stemness of osteosarcoma cells

We used a sphere formation assay to examine the effect of *MEF2C* on tumor cell stemness (**Figures 9B, C**). The results showed that the si-*MEF2C* cells formed fewer sphere (**Figure 9B**) and the size of sphere was smaller (**Figure 9C**) compared to the control group. These results can tentatively demonstrate the effect of *MEF2C* on the maintenance of stemness of CSC in osteosarcoma. Our previous analysis showed that *MEF2C* affected immune infiltration in the TME. We also verified that *MEF2C* affects the adhesion of tumor cells to the extracellular matrix by cell adhesion assays (**Figures 9D, E**).The results showed that the adhesion of si-*MEF2C* cells to the extracellular matrix such as vitronectin (**Figure 9D**) and fibronectin (**Figure 9E**) was reduced compared with the control group.

## Discussion

Recent studies have revealed that there is significant heterogeneity among tumor cells. Tumor heterogeneity is mainly manifested in several aspects such as gene expression profile, chemotherapy sensitivity, apoptosis resistance and tumorigenic ability (50). In tumor tissue, only a small proportion of tumor cells can initiate tumor formation, recurrence and metastasis, and the proportion of cells is cancer stem cells. Increasing evidence suggested that CSC plays significant roles in innate immunity, inflammation and antitumor effects (51). Nevertheless, most researches have focused on an individual cancer stem cell-associated gene or a single TME cell type, so the understanding of the overall TME infiltration characteristics mediated by the combination of multiple cancer stem cell-associated genes are not comprehensive. Exploring the combined role of multiple cancer stem cell-related genes in immune infiltration will help us further understand the role of CSC in tumor immunity and direct more effective immunotherapeutic strategies.

In this study, we identified three subtypes in osteosarcoma using the consensus clustering analysis based on 25 cancer stem cell-related genes. The three subtypes differed significantly in prognosis and had different immunophenotypes. Compared to CSC cluster B and C, CSC cluster A had more innate and adaptive immunity and stromal activation, and multiple immune-related activation pathways were also enriched in CSC cluster A. However, curiously, CSC cluster C, which also had some immune cell infiltration, was not matched for the same survival advantage. Numerous studies have revealed that the immune excluded phenotype also exhibits a large immunoctyes infiltration, but the immune cells stayed in the interstitium surrounding the tumor cell nests rather than penetrating the parenchyma of the tumor cell nests (52, 53). We therefore speculated that CSC cluster C might be an immune excluded phenotype. We also found that PDL1 expression levels were significantly elevated in cluster A, suggesting that CSC-related clusters may have potential predictive value for immunotherapy. Previous researches have revealed that TME plays critical roles in tumor progression (54). Based on the above findings, we speculated that the better prognosis of CSC cluster A might be related to its high immune infiltration. Furthermore, differentially expressed genes between three different subtypes were shown to be correlated with immune activation and CSC. These DEGs were recognized as cancer stem cell-associated genes. Similarly, three molecular subtypes were identified based on the DEGs using the consensus clustering analysis, with significant prognostic differences between the three subtypes as well as a different immune infiltration landscape. The above results again demonstrated that three distinct cancer stem cell-associated subtypes were indeed present in osteosarcoma patients and that the combined effect of multiple cancer stem cell-associated genes played a significant role in immune infiltration.

Considering the heterogeneity among tumors, we constructed a score system called CSC score to quantify the individual and thus more precisely guide the treatment of individual patients. Cancer stem cell-associated subtype characterized by abundant immune infiltration had a lower CSC score. The result suggested that the CSC score was a valuable tool to evaluate the cancer stem cell-related phenotype of individual osteosarcoma patients and to evaluate their immune infiltration. CSC is closely associated with metastasis of tumors. Therefore, we compared the differences in CSC score of patients between different clinical subgroups. The results demonstrated that the CSC score can be used to assess the metastatic status of patients. Comprehensive analyses showed that CSC score could be an independent prognostic marker for osteosarcoma. Furthermore, we observed a correlation between CSC score and PD-L1, a predictor of immune response, implying that different cancer stem cell-associated subtypes may influence the efficacy of immunotherapy. In fact, we further validated in an independent immunotherapy cohort that the CSC score may be predictive of patient response to immunotherapy as well as survival. mRNAsi is thought to be correlated with patient stemness in various cancers. Therefore, we analyzed the correlation between CSC score and mRNAsi and found that CSC score also represented the stemness of the patients to some extent. The stemness of the tumor can affect the effectiveness of drug therapy. We found that different CSC clusters have distinct sensitivities to certain small molecule drugs, thus our CSC subtypes can provide some guidance for the drug treatment of osteosarcoma patients.

Along with exploring CSC-related phenotypes, we also explored the role of individual CSC-related genes in stemness as well as in the immune microenvironment. *MYC* is encoded by the proto-oncogene family and is an essential transcription factor for the bHLH superfamily of DNA-binding proteins (55). *MYC* regulates a wide range of stem cell processes such as self-renewal and differentiation through the regulation of numerous genes (56). *MYC* has been shown to maintain the stemness of CSC in a variety of tumors, and its role in osteosarcoma stem cells has also been revealed (57–60). Meanwhile, substantial studies have focused on the effect of *MYC* on the TME. Yi et al. revealed that *MYC* amplification was associated with low immune infiltration of triple-negative breast cancer and that *MYC* may be involved in immune escape of triple-negative breast cancer by Multi-Omics Profiling (61). Stephanie et al. revealed that MYC regulates the expression of PDL1 and CD47 on the surface of tumor cells, thus modulating the tumor immune response (62). Similarly, our results showed that high *MYC* expression was negatively associated with prognosis and multiple immune infiltrating cells in osteosarcoma patients. *MEF2C* is a transcription factor which is specifically expressed in muscle and neuronal lineages and is commonly upregulated in leukemia (63). Our analysis showed that *MEF2C* expression was upregulated in tumor tissues and was related to poor patient survival outcomes. Further analyses showed that high expression of *MEF2C* was significantly and negatively related to infiltration of most antitumor lymphocytes including CD8+ T cells, NK T cells and DCs. And notably, patients with high *MEF2C* expression had higher CSC score and mRNAsi compared to those with low expression. We further confirmed the expression of *MEF2C* in osteosarcoma tissues by immunohistochemical experiments. However, the specific mechanism of its regulation of osteosarcoma stem cells and its relationship with immune infiltration still need to be explored through further experiments. Similarly, we found that high expression of *SPI1* and *EVI2B* was highly positively related to immune infiltration. These results demonstrated the importance of exploring the combined effects of multiple CSC-related genes.

In this study, we included a total of 25 cancer stem cell-related genes, and further more cancer stem cell-related genes can be included subsequently to optimize the accuracy of CSC-related subtypes. As well as the specific mechanism of the effect of some of these 25 CSCRGs on the phenotype of CSC has not been clarified, so further studies can be conducted on them subsequently. Since we used limited public datasets in this study, our findings need to be further validated in additional datasets. In this study, we initially verified the effect of *MEF2C* on the malignant phenotype of osteosarcoma by bioinformatics analysis, and verified the expression of *MEF2C* in osteosarcoma tissues by IHC. Our subsequent study will further explore the effect of *MEF2C* on the stemness of osteosarcoma through experiments. In addition, because of the relatively limited number of osteosarcoma patients currently receiving immunotherapy, this study explored the efficacy of CSC score to predict immunotherapy based on a cohort of urothelial carcinoma, and a cohort of osteosarcoma patients receiving immunotherapy would still be needed to further validate our hypothesis.

In conclusion, in this study we classified patients into three distinct subtypes based on cancer stem cell-related genes and systematically described the association between the different subtypes and the TME immunocyte infiltration characteristics. We further determined that the CSC score could be used to assess the clinical characteristics and the immune infiltration of individual osteosarcoma patients. This study provided novel ideas in identifying new tumor subtypes of osteosarcoma,guiding individualized specific therapy, and improving patient response to immunotherapy in the future.

## Data availability statement

The original contributions presented in the study are included in the article/**Supplementary Material**. Further inquiries can be directed to the corresponding author.

## Ethics statement

The studies involving human participants were reviewed and approved by Ethics Commttee of Peking University People’s Hospital. The patients/participants provided their written informed consent to participate in this study.

## Author contributions

LG and TY designed the study and wrote and revised the manuscript. WG, JN, WW, and TR acquired and analyzed the data. BW, YH, and JX wrote the manuscript. All authors read and approved the final manuscript.

## Funding

This work was supported by a grant from the National Natural Science Foundation of China (#81972513).

## Conflict of interest

The authors declare that the research was conducted in the absence of any commercial or financial relationships that could be construed as a potential conflict of interest.

## Publisher’s note

All claims expressed in this article are solely those of the authors and do not necessarily represent those of their affiliated organizations, or those of the publisher, the editors and the reviewers. Any product that may be evaluated in this article, or claim that may be made by its manufacturer, is not guaranteed or endorsed by the publisher.

## Abbreviations

AUC, Area under the curve; CSC, Cancer stem cell; CSCRGs, cancer stem cells-associated genes; DCs, Dendritic cells; DEGs, Differentially expressed genes; OS, Osteosarcoma; PCA, Principal component analysis; ROC, Receiver operating characteristic; TAMs, Tumor-associated macrophages; TME, Tumor microenvironment.
