# Supplementary material for: Molecular subtypes of osteosarcoma classified by cancer stem cell related genes define immunological cell infiltration and patient survival
Source: Front Immunol. 2022 Aug 19;13:986785. doi: 10.3389/fimmu.2022.986785 (PMC9437352; doi:10.3389/fimmu.2022.986785)
Supplement: Supplementary file 1 [file DataSheet_1.pdf]

Supplementary Material

Supplementary Figures

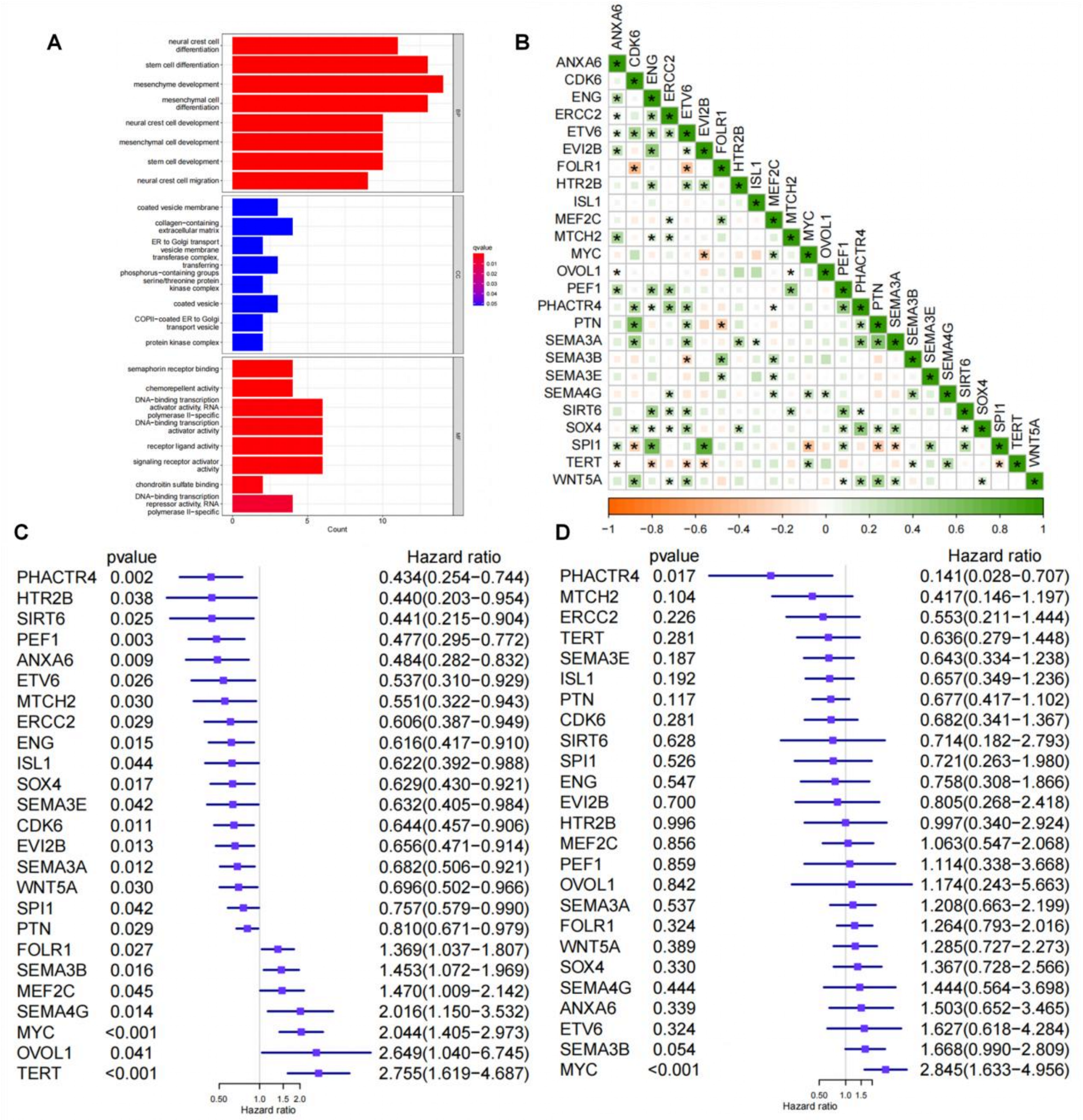

Supplementary Figure 1 Correlation and prognostic analyses of 25 CSCRGs. (A) GO enrichment analysis of the 25 genes. The x-axis indicated the amount of genes enriched. (B) The correlation between these 25 CSCRGs was calculated using the Spearman analysis. The negative correlation: yellow; positive correlation: green .*P < 0.05. (C-D) The prognostic analyses for the 25 CSCRGs in the TARGET-cohorts by univariate Cox regression (C) and multivariate Cox regression models (D).

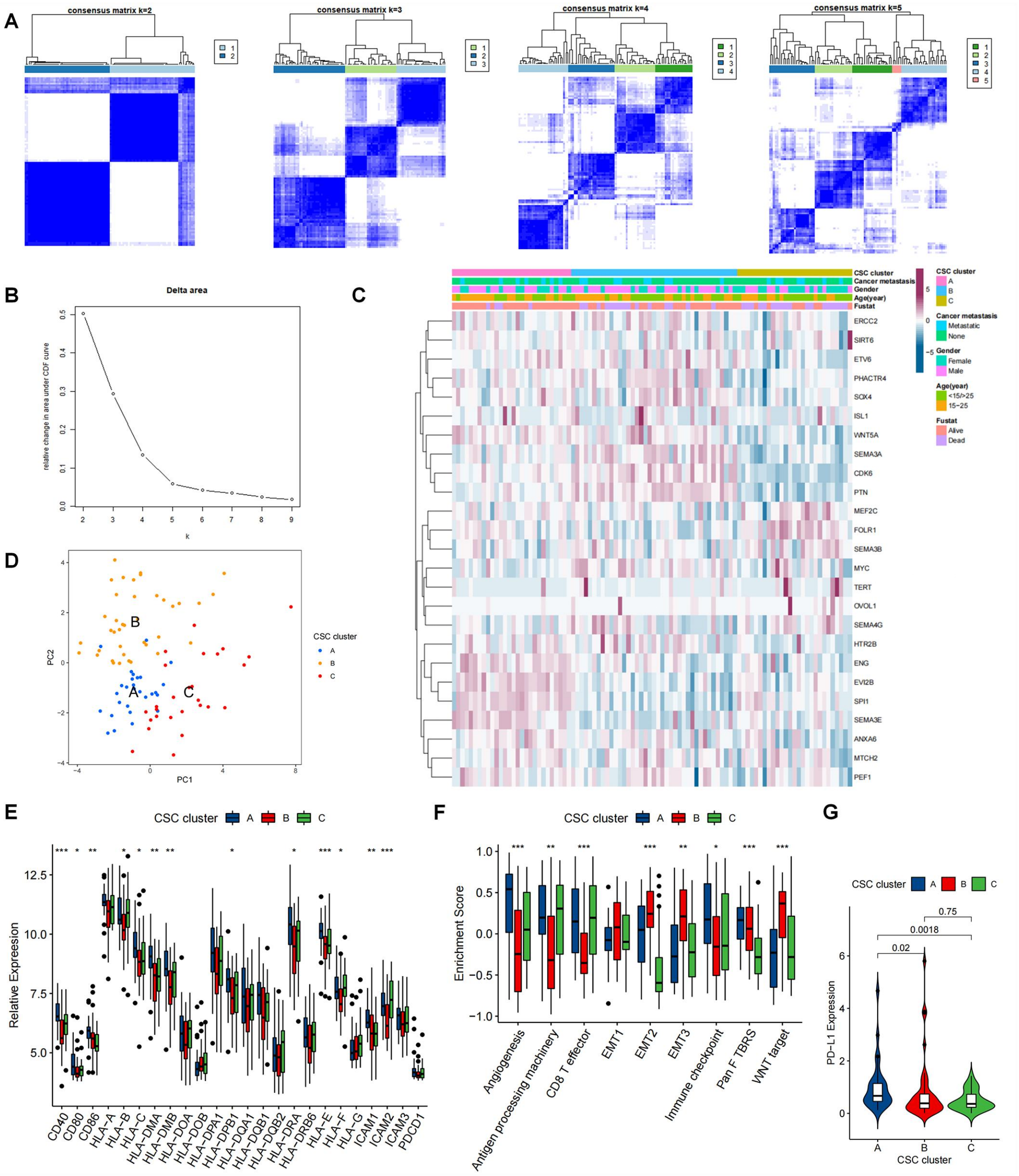

Supplementary Figure 2 Unsupervised clustering of 25 CSCRGs in the TARGET cohort. (A) Consensus matrices of the TARGET cohort for k = 2 - 5. (B)K = 3 was identified the optimal value for consensus clustering. (C) Unsupervised clustering of 25 CSCRGs in TARGET cohort. CSC cluster, age, gender, patient survival status and tumor metastasis status were annotated. Purple indicated high expression of the gene and blue indicated low expression of the gene. (D) Three CSC clusters differed significantly in the expression of 25 genes. (E) Differences in MHC molecules, co-stimulatory molecules and adhesion molecules between three CSC clusters. The bottom and top of the box indicate the 25th and 75th percentiles, and the thick line indicates the median value. (*P < 0.05; **P < 0.01; ***P < 0.001). (F) Differences in immune and stroma-related pathways between three CSC clusters. (G) Comparison of PD-L1 expression among three CSC clusters.

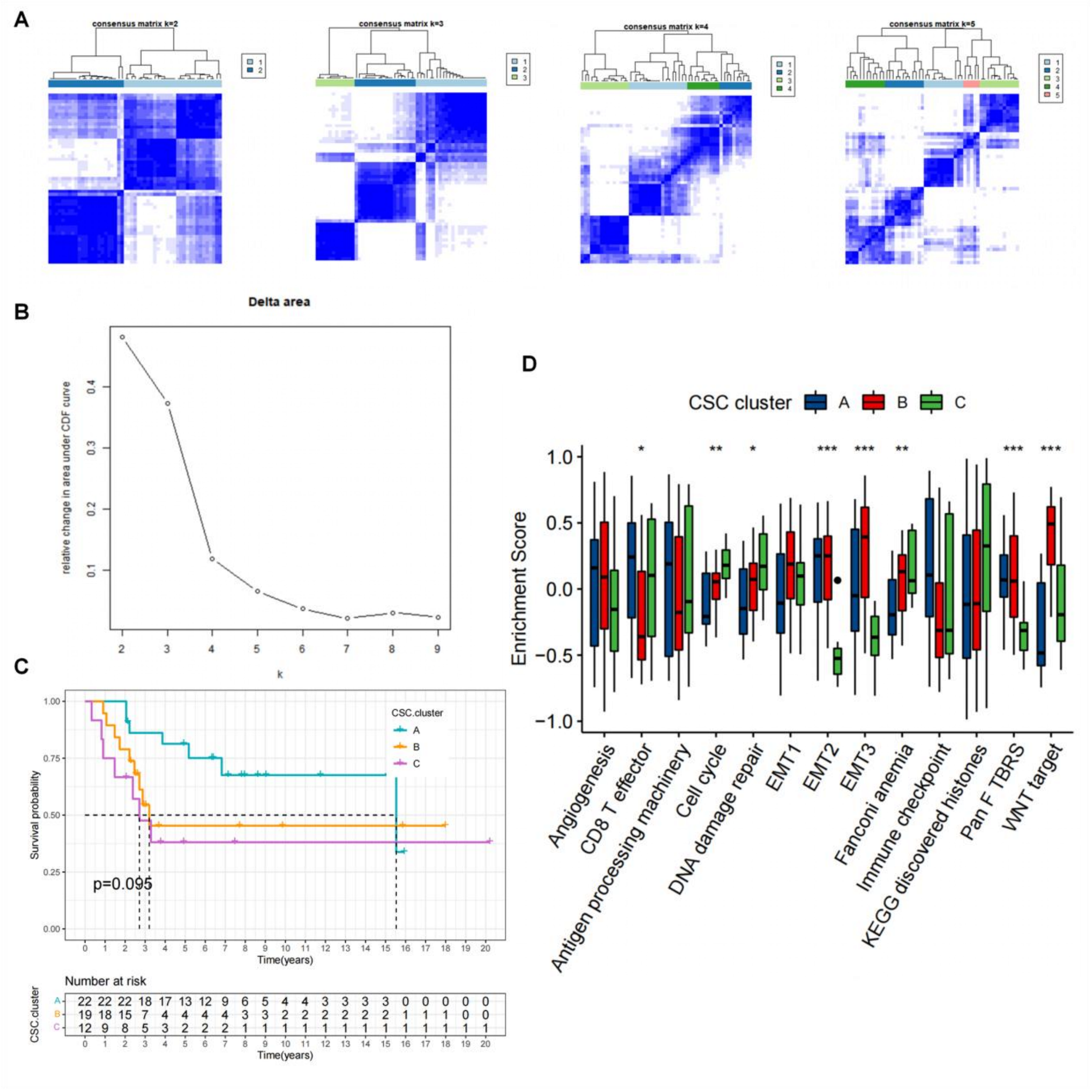

Supplementary Figure 3 Unsupervised clustering of 25 CSCRGs in the GSE21257 cohort. (A) Consensus matrices of the GSE21257 cohort for k = 2 - 5. (B) K = 3 was identified the optimal value for consensus clustering. (C) Kaplan-Meier curves of survival for 53 osteosarcoma patients in the GSE21257 cohort with different CSC clusters, including 22 cases in CSC cluster A, 19 cases in CSC cluster B, and 12 cases in CSC cluster C. (D) Differences in immune and stroma-related pathways between three CSC clusters.

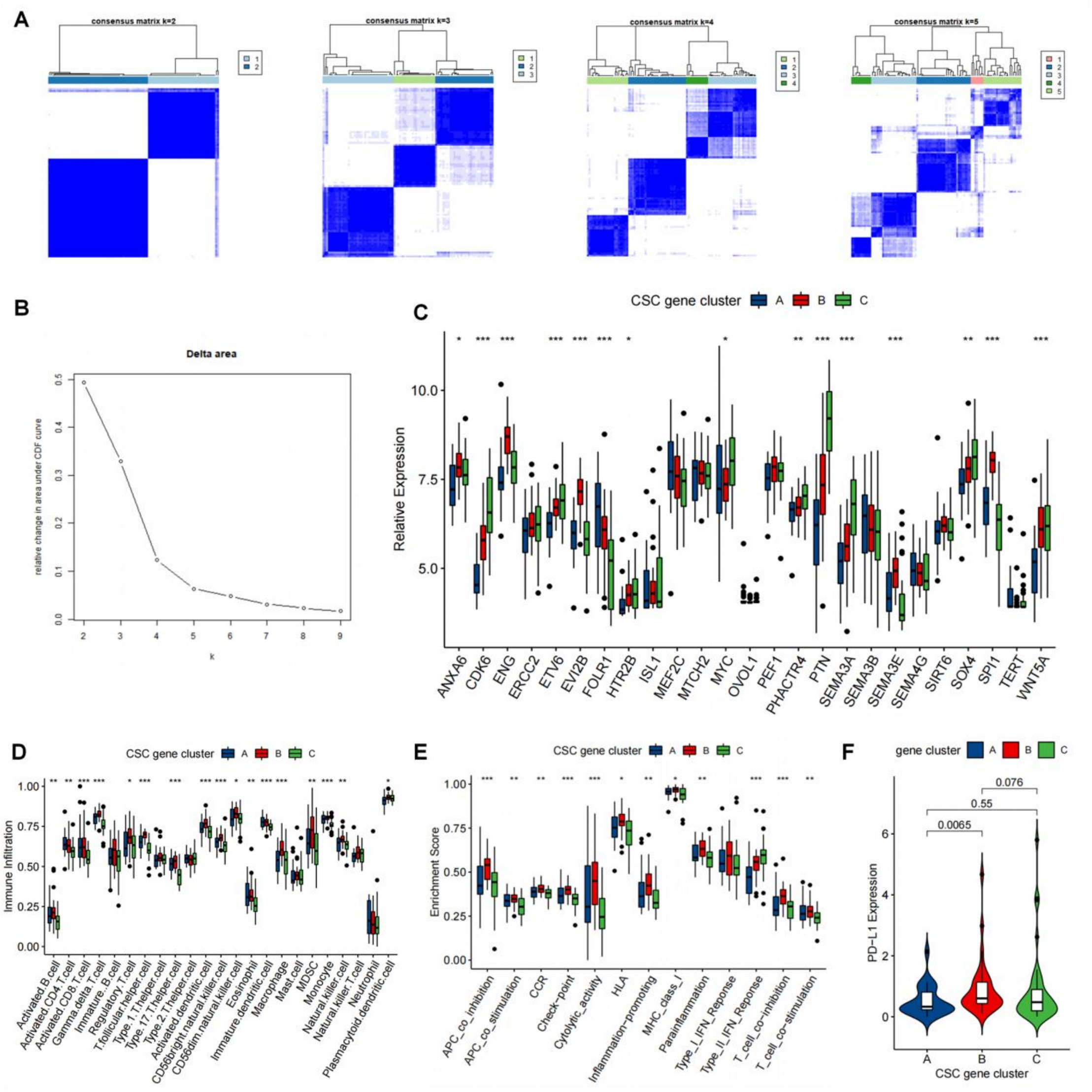

Supplementary Figure 4 Unsupervised clustering of 104 CSC-related genes in the TARGET cohort. (A) Consensus matrices of the TARGET cohort for k = 2 - 5. (B) K = 3 was identified the optimal value for consensus clustering. (C) Comparison of 25 CSCRGs between three CSC gene clusters. (D) Difference in the abundance of each immunocyte between three CSC gene clusters. (E) Differences in the immune-related functions between three CSC gene clusters. (F) Comparison of PD-L1 expression between three CSC gene clusters.

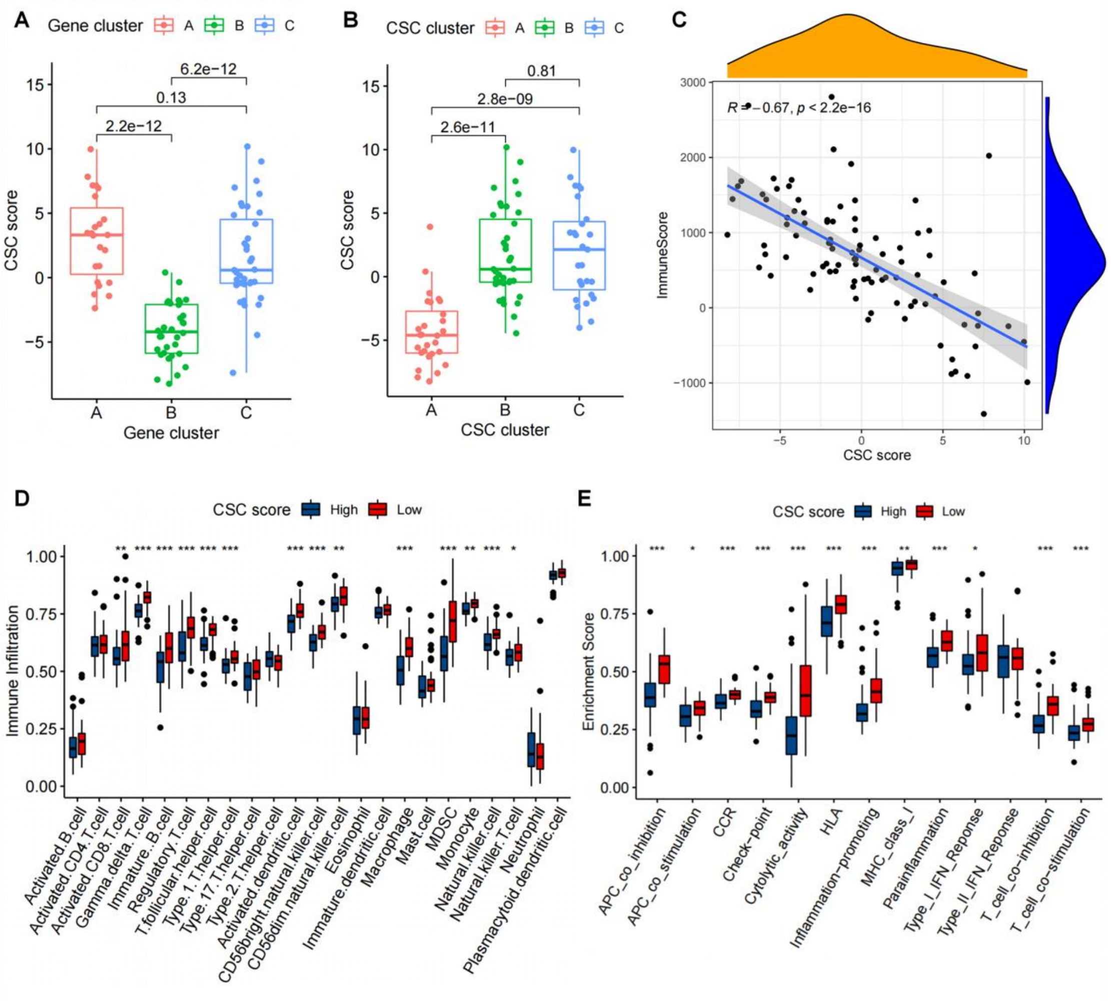

Supplementary Figure 5 CSC score associated with TME infiltration and survival outcome. (A) Comparison of CSC scores among three CSC gene clusters. (B) Comparison of CSC scores among three CSC clusters. (C) CSC score was also significantly negatively correlated with immune score (r = -0.67, P < 0.05). (D) Difference in the abundance of each immunocyte between high and low CSC score subgroups. (E) Differences in the immune-related functions between high and low CSC score subgroups.

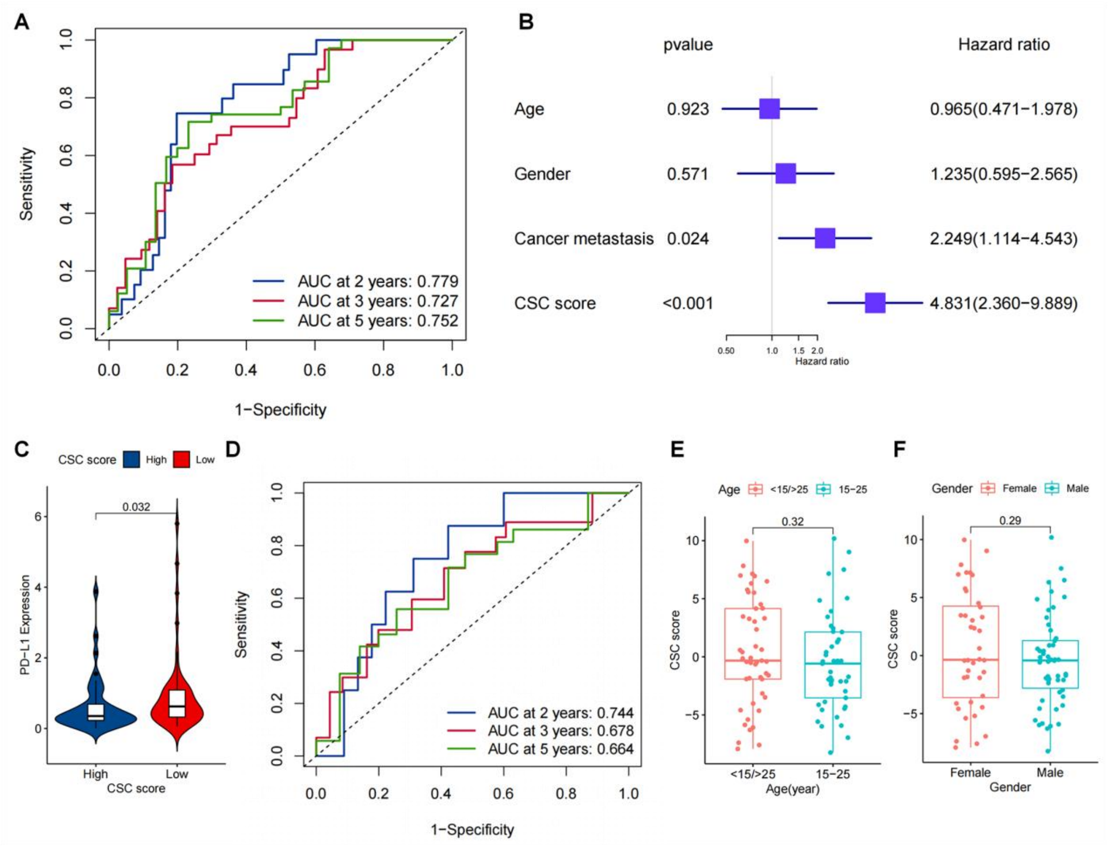

Supplementary Figure 6 Independent validation of CSC score associated with survival outcome. (A) The predictive value of CSC score in TARGET cohorts. (B) Multivariate Cox regression model analysis taking into account multiple clinical traits of patients. The length of the horizontal line represent the 95% confidence interval. (C) Comparison of PD-L1 expression between high and low CSC score subgroups. (D) The predictive value of CSC score in GSE21257 cohorts. (E) Comparison of CSC scores between different age groups. (F)Comparison of CSC scores between different gender groups.

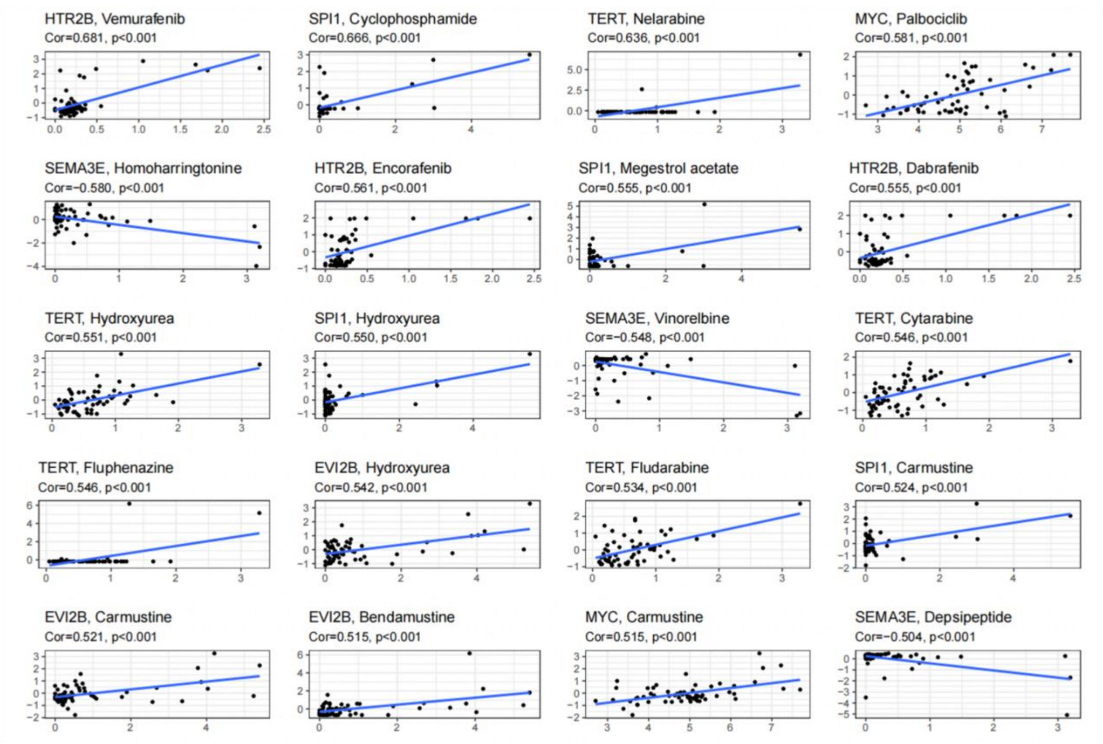

Supplementary Figure 7 Correlation of 25 CSCRGs expression level and IC50 of different drugs.
